# Remarks from Editor-in-Chief on the celebration of the 65th anniversary of Acta Biochimica et Biophysica Sinica

**DOI:** 10.3724/abbs.2023116

**Published:** 2023-06-20

**Authors:** Jianping Ding

**Affiliations:** Editor-in-Chief; Acta Biochimica et Biophysica SinicaJune 2023

Dear colleagues and friends,

It is with great joy that we celebrate the 65th anniversary of the founding of Acta Biochimica et Biophysica Sinica (ABBS). Looking back at the development of ABBS, we are filled with immense pride and gratitude.

ABBS is a professional academic journal supervised by the Chinese Academy of Sciences (CAS) and sponsored by the Center of Excellence in Molecular Cell Science (CEMCS, Shanghai Institute of Biochemistry and Cell Biology), which has evolved in the last 65 years from a Chinese vernacular journal to an English journal with international impact. The Journal was founded as Acta Biochimica Sinica in 1958 by Professor Yinglai Wang, who served as the first Editor-in-Chief. In 1961, the Journal was renamed as Acta Biochimica et Biophysica Sinica (ABBS), with the aim of developing into an authoritative journal in the research field of biochemistry and biophysics in China. In the early years of its establishment, the Journal published a number of important scientific achievements, such as the preliminary research results on synthetic crystalline bovine insulin between 1961 and 1964, and a series of research papers on “Kinetics of irreversible modification of enzyme activity” by Professor Chen-Lu Tsou in 1965. These scholarships witnessed the beginning and development of biochemistry in China and promoted the progress of biochemistry and biophysics in China.

In 1998, as one of the most influential academic journals in China, ABBS became one of the first Chinese vernacular journals to be indexed in Medline database and SCIE database, which was an important milestone for the journal. To adapt to the rapid progress of scientific research in China and to enhance further international exchange, ABBS was converted to a monthly English journal in 2004. This change significantly promoted the international dissemination of Chinese scientific research works. In 2005, ABBS established a partnership with Blackwell Publishing Asia Ltd, which opened the mode of global publication and distribution for the journal, and took the lead in starting the online version of the journal. ABBS became one of the first few journals in China to explore international cooperation in publishing and marketing. In 2009, ABBS set up a cooperative relationship with Oxford University Press and published the journal simultaneously in both print and electronic versions. In doing so, the subscribers of ABBS have covered most of the developed countries and regions including Europe, the United States, and Japan, as well as more than 30 developing countries. Through these international collaborations, ABBS has achieved tremendous development and accelerated the international dissemination of Chinese research works.

The Open Access (OA) publishing model has brought new opportunities for the prosperity of academic journals, and also provided a possibility for Chinese scientific and technological journals to explore the way to grow and develop independently. In 2022, ABBS, as the first academic journal, started the journey of returning back to China to publish independently. With the SciEngine, a full-process digital publishing platform provided by Science Press, ABBS started OA publishing and distribution by itself. The Journal has accomplished the localization of the whole publishing process covering content production, publication, distribution, and data storage, and kept the trend of stable development. This paved a new path for the development of Chinese scientific journals.

Over the past 65 years, ABBS has been adhering to the missions of serving the scientific community and providing a professional, friendly and high-quality publication platform for scientific researchers to publicize and disseminate their works. At present, ABBS has grown into a professional academic journal with international influence. In the last 5 years, the Journal has published more than 940 scientific papers with nearly 4000 authors from more than 600 research institutions in 25 countries and regions worldwide; furthermore, the research content of the papers has covered hot research areas such as protein science, RNA regulation, epigenetics, DNA damage repair, tumor metabolism and immunity, enzyme structure and function regulation, cell signaling pathways, and cell death and apoptosis, and much more. The Journal has reached over 300,000 views and downloads per year, and 60% of the readers are international researchers.

To meet the new opportunities and challenges in the future, ABBS will continue to commit to the original missions of the journal: to actively promote the publication and dissemination of cutting-edge research works in biochemistry and biophysics, and to provide a high-level, high-quality professional publication platform with better services and supports to the scientific community.

On the occasion of the 65th anniversary of ABBS, as the current Editor-in-Chief of ABBS, I would like to express my sincere gratitude to CAS and CEMCS for their full supports, and to the former Editor-in-Chiefs, the members of the past and current Editorial Boards, all the reviewers, and all the researchers who have supported and cared about the development of ABBS for their selfless dedication, tireless efforts, and valuable contributions. Only because of you, ABBS has flourished over the past 65 years. I sincerely hope that ABBS will continue to receive your fondness and support, and I look forward to working together to further promote the development of biochemistry and biophysics in China.

To celebrate ABBS’ 65th anniversary, ABBS has organized and published two special issues: the current issue focusing on cancer biology and the next issue focusing on liquid-liquid separation. These review articles cover the main research hotspots in these fields, summarizing the research progresses in recent years and providing the authors’ perspectives on future developmental directions. We hope that readers will benefit from these two special issues in their future research work.

